# Review of the 25^th ^annual scientific meeting of the International Society for Biological Therapy of Cancer

**DOI:** 10.1186/1479-5876-9-60

**Published:** 2011-05-12

**Authors:** James M Balwit, Pawel Kalinski, Vernon K Sondak, Pierre G Coulie, Elizabeth M Jaffee, Thomas F Gajewski, Francesco M Marincola

**Affiliations:** 1 Society for Immunotherapy of Cancer, Milwaukee, WI, USA; 2 University of Pittsburgh Cancer Institute, Pittsburgh, PA, USA; 3 Department of Cutaneous Oncology, Moffitt Cancer Center, and Departments of Oncologic Sciences and Surgery, University of South Florida, Tampa, FL, USA; 4 de Duve Institute and Université Catholique de Louvain, Brussels, Belgium; 5 Department of Oncology, the Skip Viragh Pancreatic Cancer Center, Johns Hopkins University, Baltimore, MD, USA; 6 University of Chicago, Department of Pathology and Department of Medicine, Section of Hematology/Oncology, Chicago, IL, USA; 7 Infectious Disease and Immunogenetics Section (IDIS), Dept. of Translation Medicine, Clinical Center, and Center for Human Immunology (CHI), National Institutes of Health, Bethesda, MD, USA

## Abstract

Led by key opinion leaders in the field, the 25th Annual Meeting of the International Society for Biological Therapy of Cancer (iSBTc, recently renamed the Society for Immunotherapy of Cancer, SITC) provided a scientific platform for ~500 attendees to exchange cutting-edge information on basic, clinical, and translational research in cancer immunology and immunotherapy. The meeting included keynote addresses on checkpoint blockade in cancer therapy and recent advances in therapeutic vaccination against cancer induced by Human Papilloma Virus 16. Participants from 29 countries interacted through oral presentations, panel discussions, and posters on topics that included dendritic cells and cancer, targeted therapeutics and immunotherapy, innate/adaptive immune interplay in cancer, clinical trial endpoints, vaccine combinations, countering negative regulation, immune cell trafficking to tumor microenvironment, and adoptive T cell transfer. In addition to the 50 oral presentations and >180 posters on these topics, a new SITC/iSBTc initiative to create evidence-based Cancer Immunotherapy Guidelines was announced. The SITC/iSBTc Biomarkers Taskforce announced the release of recommendations on immunotherapy biomarkers and a highly successful symposium on Immuno-Oncology Biomarkers that took place on the campus of the National Institutes of Health (NIH) immediately prior to the Annual Meeting. At the Annual Meeting, the NIH took the opportunity to publicly announce the award of the U01 grant that will fund the Cancer Immunotherapy Trials Network (CITN). In summary, the Annual Meeting gathered clinicians and scientists from academia, industry, and regulatory agencies from around the globe to interact and exchange important scientific advances related to tumor immunobiology and cancer immunotherapy.

## Guiding Cancer Immunotherapy from Bench to Bedside

The opening scientific presentation--the Richard V. Smalley, MD Memorial Lectureship--was delivered by James P. Allison, PhD (Memorial Sloan-Kettering Cancer Center), a pioneer in the development of CTLA-4 blockade. Dr. Allison presented data on new insights and opportunities in checkpoint blockade. He outlined a number of key points to be addressed for further clinical development of anti-CTLA-4, including better understanding cellular and molecular mechanisms, identifying characteristics that distinguish responders from non-responders, and determining the best conventional therapies or vaccines to combine with CTLA-4 blockade to improve outcomes in patients with cancer.

## Dendritic Cells in Cancer

Carl G. Figdor, PhD (Nijmegen Centre for Molecular Life Sciences) and Pawel Kalinski, MD, PhD (University of Pittsburg Cancer Institute) co-chaired the first plenary session on dendritic cells (DCs) and cancer. Dr. Figdor addressed achievements, obstacles, and future perspectives of DC vaccination. Progress in active immunotherapy of prostate cancer with the autologous cellular immunotherapy Sipuleucel-T was reported by David L. Urdal (Dendreon Corporation). Bart Neyns, MD, PhD (Universitair Ziekenhuis Brussel) presented on therapeutic vaccination with mRNA-electroporated autologous DCs in patients with advanced melanoma. Andrew N. Cornforth, PhD (Hoag Memorial Hospital Cancer Center) presented data that demonstrate that resistance to the proapoptotic effects of IFN-γ on melanoma cells used in patient-specific DC immunotherapy is associated with improved overall survival. Research conducted and reported by Jamie L. Harden (State University of New York, University at Buffalo) suggests that IFN-gamma is central to both immunogenic and tolerogenic properties of DCs after IL-12 and GM-CSF microsphere treatment. Dr. Kalinski concluded the session with a presentation on polarized (high IL-2 producing) DCs in the immunotherapy of established cancer, in which he reviewed data indicating that αDC1s preferentially attract naïve effector and memory T cells rather than Tregs and that this subset is more efficient in inducing tumor-specific CTLs than standard DCs. Dr. Kalinski presented results that demonstrate that αDC1s induce CTL effector functions and responsiveness to tumor-produced chemokines in naïve and resting memory CD8^+ ^T cells. Moreover, vaccines from αDC1s can be generated from patients with a large variety of cancers.

## Targeted Therapeutics and Immunotherapy

Mary L. Disis, MD (University of Washington) and Keiran S. Smalley, PhD (H. Lee Moffitt Cancer & Research Institute) co-chaired a session on targeted therapeutics and immunotherapy. Dr. Disis started the session with a presentation on immune modulation of breast cancer. She presented data from animal models in which the development of breast cancer was inhibited by immunization against a limited number of biologically relevant antigens. While subclinical disease may limit protection, combination chemoprevention may enhance vaccine efficacy. Dr. Disis concluded that vaccine strategies may benefit from targeting proteins involved in oncogenesis.

Dr. Smalley reported on overcoming BRAF inhibitor resistance in melanoma, noting that pERK recovers with higher doses of PLX4720, potentially limiting the drug's effects. Low-level pERK signaling appears important for a small subpopulation of cells to escape therapy. Dual BRAF-MEK inhibition, he concluded, may limit escaping cells.

Kim A. Margolin, MD (University of Washington, Seattle Cancer Care Alliance) reported on results from the "SELECT" trial of high-dose IL-2 treatment of patients with metastatic renal cell carcinoma. In this trial, the response rate with high-dose IL-2 was significantly higher than historically achieved, likely related to differences in patient selection. Dr. Margolin noted that clinical and pathologic characteristics may help identify patients who are unlikely to benefit from high-dose IL-2 treatment. Pathology review and staining for CA-IX in this trial, however, did not improve selection of patients who benefited from treatment.

Chunsheng Li, PhD (Ovarian Cancer Research Center, University of Pennsylvania) discussed *in vivo *modeling and detection of ovarian cancer vascular marker TEM1, which was observed in 99% of ovarian cancer specimens studied. This vascular model, Dr. Li suggested, can serve as a platform both for studying the function of tumor vascular markers and for testing new diagnostics and therapeutic agents against tumor vasculature *in vivo*.

## Innate/Adaptive Immune Interplay in Cancer

Vincenzo Cerundolo, MD, PhD (University of Oxford, Institute of Molecular Medicine) and Laurence Zitvogel, MD, PhD (Institute Gustave Roussy) co-chaired a session on the interplay between innate and adaptive immunity in cancer. Dr. Cerundolo addressed the role of invariant NKT (iNKT) cells at the interface of innate and adaptive immunity, noting that incubating neutrophils with serum amyloid A (SAA), which is secreted by a large proportion of tumor-associated macrophages (TAM) and primary melanoma cells, facilitates their cross-talk with iNKT cells, leads to iNKT cell activation and reduces IL-10 secretion. Dr. Cerundolo highlighted the need to further explore and exploit the capacity of iNKT cells to reduce the immunosuppressive activity of neutrophils in melanoma.

Khashayarsha Khazaie, PhD, DSc (Northwestern University, Robert Lurie Comprehensive Cancer Center) presented on the role of Tregs in colon cancer, highlighting observations that were consistent with Tregs playing an anti-inflammatory and protective role in colorectal cancer, which can be compromised through interactions with mast cells over the course of the disease. These results suggest that cross-talk between mast cells and Tregs determines the level of inflammation in colorectal cancer.

Bethany Mundy (The Ohio State University) discussed myeloid-derived suppressor cells (MDSCs) and decreased IFN responsiveness in tumor-bearing mice, demonstrating that MDSCs inhibited IFN response via nitration of STAT1 in tumor-bearing mice and that MDSC depletion from tumor-bearing mice restored the IFN response.

Laurence Zitvogel, MD, PhD (Institute Gustave Roussy, INSERM France) presented on NKp30 isoforms as a novel predictor of response to tyrosine kinase inhibitors. Dr. Zitvogel reviewed functional differences in the isoforms: DC/NK or NK/tumor cross-talk induces IL-10 with the NKp30c isoform and IFNγ with isoforms NKp30a and NKp30b. Clustering of the NKp30 transcriptional profiles could be used to discriminate between responders and non- responders to Gleevec for gastrointestinal stromal tumors, with less than 20% of profile A responders experiencing relapse, versus >40% relapse among non-responders with profile B.

## Clinical Trial Endpoints

Vernon K. Sondak, MD (H. Lee Moffitt Cancer Center) and F. Stephen Hodi, MD (Dana-Farber Cancer Institute) co-chaired a session on clinical trial endpoints. Dr. Sondak reviewed endpoints and clinical trial designs in historical studies on melanoma and recent results with ipilimumab. He emphasized that progress in the systemic therapy of metastatic melanoma requires well-designed, well-executed phase III trials using agents appropriately selected in phase II studies, and that eligibility criteria, patient selection and study size account for a large percentage of the variation in outcomes in phase II trials. He suggested that six-month progression-free survival (PFS) and 12-month overall survival (OS) may be better "selection" endpoints for phase II trials in melanoma than objective response or median survival. New trial designs, such as adaptive randomization, and careful and individualized selection of endpoints are going to be necessary to evaluate the increasing number of promising agents in melanoma and other malignancies.

Dr. Hodi presented on immune-related response criteria (irRC) noting the challenges of cancer immunotherapy clinical trial endpoints, including the observation that activation and proliferation of immune cell begins early and that immunotherapy effects on tumors may take weeks to months to develop. Moreover, effects of immunotherapy on survival may take years to assess. These responses are further complicated by variability in cellular immune assays and the potential for slow onset and late clinical effects from immunotherapy. Using CTLA-4 blockade with ipilimumab as a platform, Dr. Hodi discussed the modification of anti-tumor response endpoints to incorporate late onset of clinical activity (i.e., response after progression), which is common with CTLA-4 blockade in melanoma.

Lawrence Fong, MD (University of California, San Francisco) focused on defining response in prostate cancer immunotherapy. While OS remains the definitive outcome for defining clinical efficacy in prostate cancer, it is not be feasible for early trial development. Immunotherapies that induce clinical responses in prostate cancer provide the opportunity to help redefine meaningful immune "responses." Dr. Fong concluded that clinical endpoints that allow for early progression are being used in prostate cancer trials (e.g., Prostate Cancer Clinical Trials Working Group) and should be applied to immunotherapy trials in prostate cancer. Moreover, immune correlates need to be validated in larger trials (with clinical benefit), including Phase 3 clinical trials that are followed for overall survival.

Annick Van den Abbeele, MD (Dana-Farber Cancer Institute) discussed a new paradigm in cancer imaging (i.e., metabolic imaging). Dr. Van den Abbeele noted that in the future, cancer imaging will not rely solely on measurement of tumor size, but rather will employ a multiparametric approach designed to interrogate cellular and molecular events in tumor cells, the immune system, and within the tumor microenvironment. Future cancer imaging will provide information on the mechanism of action of novel therapies and will provide quantitative, noninvasive, pharmacodynamic information on tumor metabolism.

## Cancer Immunotherapy Guidelines: A New iSBTc/SITC Initiative

In a special session, Howard L. Kaufman, MD (Rush University Medical Center) announced a new iSBTc/SITC initiative: the development of cancer immunotherapy guidelines. These guidelines will provide evidence-based criteria for defining the clinical indications for immunotherapy and provide suggestions for when and how to use immunotherapy in patients with cancer. A multi-disciplinary task force is collecting and reviewing current literature to make recommendations. These recommendations will be developed into a manuscript and the guidelines will be made available to the public through the society's web site.

## Immunotherapy of High Risk HPV Infections

Cornelius J.M. Melief, MD, PhD (Leiden University Medical Center) provided the second keynote address on therapeutic vaccination against HPV16-induced disease. Dr. Melief presented data that demonstrate that short peptide vaccines are not effective in therapeutic vaccines against HPV16-induced cancer; long peptide vaccines that harbor both CD4 and CD8 T cell epitopes and require DC processing, however, are quite efficient. Further improvements are possible by adding TLR ligands or by conjugating TLR ligands to the long peptides. Dr. Melief suggested development of combination treatment with long peptide vaccination, immunogenic chemotherapy and inhibitors of checkpoint control (CTLA-4 blocker, PD-1, PD-L1 blockers) for maximally effective cancer treatment. He also suggested local delivery of mAb treatments in slow release formulation close to tumor-draining lymph nodes to reduce toxicity.

## Vaccine Combinations

Pierre Coulie, MD, PhD (de Duve Institute and Université Catholique de Louvain) and Victor H. Engelhard, PhD (University of Virginia School of Medicine) co-chaired a session on vaccine combinations. Dr. Coulie addressed the numbers and functions of lymphocytes in human melanoma metastases. Vaccine trials in metastatic melanoma have used a variety of tumor-specific Ag administered as peptides, proteins, peptide-pulsed DCs, and recombinant poxviruses. Among patients who displayed tumor regression (10% - 20%), the number of anti-vaccine CTLs was low. Further analysis indicated that tumor regression was linked with activation of CTLs that recognize other tumor-specific antigens not included in the vaccine, and that some of these CTLs were in the blood and tumors prior to vaccination. New CTLs appeared following vaccination. These included both new clones against antigens targeted prior to vaccine administration (i.e., clonal spreading) and new clones against previously ignored antigens (i.e., antigen spreading). Dr. Coulie suggested that melanoma patients spontaneously mount anti-tumor CTL responses that become inefficient at rejecting the tumor due to local immunosuppression or reduced tumor antigen expression. Vaccination may activate a small number of anti-vaccine CTLs, which upon trafficking to the tumor, may relieve suppression and promote activation of many other anti-tumor CTLs that contribute to tumor regression.

Victor H. Engelhard, PhD (University of Virginia School of Medicine) discussed immunological proteomics, the identification of phosphorylated peptide antigens displayed on cancer cells, and prospects for their use as immunotherapeutics. Dr. Engelhard noted that few of the currently identified melanoma Ags are from proteins associated with transformation and/or metastasis. Identification of Ags from proteins associated with control of cell growth, survival or metastasis may be useful because alteration of these essential proteins associated with immune evasion may comprise survival of the tumor cell.

Phosphorylation of signaling molecules regulates activation and proliferation of many cancers. Dr. Engelhard and colleagues have developed a strategy to identify MHC-associated peptides modified by intracellular phosphorylation using mass spectrometry. This has allowed isolation of a small number of peptides that are associated with cellular transformation or metastasis. As a candidate for immunotherapy, the phosphopeptide should be expressed on melanomas from multiple patients, with little or no expression on normal cells. It should be associated with an aspect of malignancy and must be immunogenic. Ideally, it would be expressed on other kinds of cancer cells as well. Phosphopeptides associated with melanoma are immunogenic. Phosphopeptide-specific CD8 T cells differentiate between the phosphorylated and non-phosphorylated peptides and recognize melanoma cells. Dr. Engelhard and colleagues have recently demonstrated that the phosphate moiety is readily accessible for direct interaction with the TCR and the MHC molecule, increasing the peptides affinity and modifying its conformation. Thus, phosphorylation can generate new Ags. MHC-restricted phosphopeptides warrant further investigation as potential targets for melanoma immunotherapy.

Hideho Okada, MD, PhD (University of Pittsburgh Cancer Institute) presented on the induction of CD8^+ ^T cell responses against novel glioma-associated antigen (GAAs) peptides and clinical activity by vaccinations with αDC1s and Poly-ICLC in patients with recurrent malignant glioma. The first four vaccines induced positive immune responses against at least one of the targeted GAAs in 11 of 19 patients, with booster vaccination leading to positive responses in an additional four patients. Type 1 cytokines and chemokines were up-regulated and eight patients experienced progression free status ≥12 months; one patient with recurrent glioblastoma multiforme achieved sustained clinical response. IL-12 production by αDC1s correlated positively with PFS.

Jedd D. Wolchok, MD, PhD (Memorial Sloan-Kettering Cancer Center) discussed endogenous and exogenous vaccination in the context of immunologic checkpoint blockade. Dr. Wolchok reviewed data that demonstrate that CTLA-4 blockade with ipilimumab results in prolonged survival of patients with refractory melanoma. Moreover, clinical response has been associated with changes in absolute lymphocyte count, NY-ESO-1 immunity, and induction of ICOS expression on CD4^+ ^T cells. These responses require prospective evaluation in ongoing clinical trials. Indeed, the tumor microenvironment remains fertile ground to study the mechanism underlying immunologic checkpoint blockade.

Saskia J. Santegoets, PhD (VU University Medical Center) presented on T cell activation, PSMA seroconversion and increased Th17 rates associated with favorable clinical outcome in prostate cancer patients treated with prostate GVAX and anti-CTLA-4 immunotherapy. Dr. Santegoets reported that treatment was associated with induction of T cell activation and that rates of Tregs increased in patients with progressive disease (PD), but not in those with partial response (PR) or stable disease (SD). Indeed, elevations in Treg levels were associated with reduced survival. Treatment with GVAX/ipilimumab led to Th17/Th2 polarization, with Th17 induction coinciding with the onset of immune-related adverse events and a decline in PSA, and induction of Th2 associated with better OS. Dr. Santegoets reported serological PSMA responses associated with treatment and that PSMA seroconversion was associated with improved OS.

Willem W. Overwijk, PhD (University of Texas, MD Anderson Cancer Center) presented data suggesting that vaccine sites can serve as sinks and become a "graveyard" for tumor-specific T cells. Dr. Overwijk's results indicate that oil-based, long-lived vaccine formulations activate T cells, but eventually tolerize the cells. While this tolerance can be overcome with additional adjuvants, oil-based vaccines tend to sequester T cells at the vaccine site and limit T cell accumulation in the tumor. Thus, long-lived vaccines can induce sub-optimal anti-tumor immunity. Water-based, short-lived vaccine formulations require additional adjuvants to activate T cells, but do not tend to sequester T cells at the vaccine site. Consequently, these formulations allow T cell accumulation in the tumor and may have greater therapeutic efficacy than long-lived formulations. Thus short-lived vaccine formulations warrant further evaluation.

Antoni Ribas, MD (University of California, Los Angeles) presented final efficacy results of A3671009, a phase III study of tremelimumab versus chemotherapy (dacarbazine or temozolomide) in first-line patients with unresectable melanoma. Compared with chemotherapy, tremelimumab resulted in a nonsignificant improvement in survival. The duration of first objective tumor responses to tremelimumab was significantly longer than responses to chemotherapy. A low baseline level of C reactive protein (CRP) and a baseline absolute lymphocyte count in the normal range selected for patients with higher tumor response rate and better survival outcome with tremelimumab compared with chemotherapy, which may reflect an interaction between the tumor microenvironment, tumor inflammation, and an adaptive immune response.

## Presidential Abstract Session

Based on the high quality of the data as reviewed by a special committee, four abstracts from young investigators were selected for presentation during the presidential abstract session, chaired by iSBTc/SITC President Bernard A. Fox, PhD (Earle A. Chiles Research Institute). Robbert Spaapen, PhD (University of Chicago) presented results that indicate that interferon-β secretion in the tumor microenvironment can cause potent tumor control through host cells independently from adaptive immunity. Results reviewed by Michael A. Curran, PhD (Memorial Sloan-Kettering Cancer Center) demonstrate that 4-1BB activation induces the master-regulator EOMES and a broad-spectrum Th1 phenotype, which synergizes with CTLA-4 blockade to reject B16 melanoma. Evripidis Lanitis, BS (University of Pennsylvania) indicated that ovarian cancer cells ubiquitously express HER-2, and that even very low levels of HER-2 can lead to specific recognition by genetically redirected T cells. Chao Ma, MS (California Institute of Technology) presented on large-scale profiling of circulating serum markers, single cell polyfunctionality and antigen diversity of T cell response against melanoma.

## Countering Negative Regulation

Pierre van der Bruggen, PhD (Ludwig Institute for Cancer Research) and Weiping Zou, MD, PhD (University of Michigan) co-chaired a session exploring approaches to counter negative regulation of anti-tumor T cell responses. Dr. van der Bruggen addressed the possibility of correcting the impaired function of human TILs. While the identification of human tumor-specific Ags has allowed the development of numerous cancer vaccine strategies, these approaches have shown low clinical efficacy in melanoma patients, in part because of the immunosuppressive environment of the tumor. In contrast to circulating CD8^+ ^blood cells, CD8^+ ^TILs demonstrated impaired *ex vivo *IFN-γ secretion due to decreased TCR mobility upon trapping within a galectin-3 clustered glycoprotein lattice. IFN-γ secretion could be restored by treating the TILs with a galectin ligand N-acetyllactosamine (LacNAc). Dr. van der Bruggen discussed the hypothesis that activation of TILs may modify the N-glycosylation pathway and the structure of surface expressed N-glycans to enhance binding of galectin-3, which is abundant in many solid tumors, thereby impairing TCR mobility. Soluble competitive ligands (e.g., anti-galectin-3 Ab) may restore TCR mobility and boost IFN-γ secretion. A plant-derived polysaccharide in clinical development has the capacity to detach galectin-3 from CD8^+ ^and CD4^+ ^TILs and increase their IFN-γ production *ex vivo*. In a mouse model, half of tumor-bearing mice that were vaccinated with tumor Ag and received the polysaccharide rejected the tumor, whereas the polysaccharide alone had no benefit, suggesting the value of exploring combinations of galectin-3 ligands with therapeutic cancer vaccines in clinical trials.

Leisha A. Emens, MD, PhD (Johns Hopkins University School of Medicine) presented results indicating that the multikinase inhibitor sorafenib reverses the suppression of IL-12 and enhancement of IL-10 by PGE_2 _in murine macrophages. Further, sorafenib appears to inhibit PGE_2_-induced IL-10 secretion, indirectly preventing STAT3 activation. Moreover, in murine macrophages, sorafenib inhibits p38 MAPK activation, thereby preventing MSK1 activation. Dr. Emens concluded that sorafenib impacts the cytokine profile of macrophages by an ERK-independent mechanism.

Michael P. Gustafson, PhD (Mayo Clinic) discussed the loss of HLA-DR expression on CD14^+ ^cells as a common marker of immunosuppression in cancer patients. Dr. Gustafson suggested that immunophenotyping (via flow cytometry and multiparameter analysis) will be extremely important in characterizing patients' baseline immunity. He argued that a bioinformatics approach will likely reveal new relationships among immune cells. He noted that CD14^+^HLA-DR^lo/neg ^monocytes are elevated in all cancer types analyzed and that these monocytes inhibit T cell proliferation and cannot fully mature into potent DCs. Thus, he suggested that a combination of CD14^+^HLA-DR^lo/neg ^monocytes and other phenotypes may be prognostic, independent of therapy, and may help identify potential responders/non-responders among patients receiving cancer vaccines or other immunotherapies.

Weiping Zou, MD, PhD (University of Michigan) presented on polyfunctional Tregs in the microenvironments of cancer and inflammation. While Foxp3^+^CD4^+ ^regulatory T cells have been shown to inhibit immune responses and to temper inflammation, IL-17^+^CD4^+ ^T (Th17) cells have been shown to mediate inflammatory processes of autoimmune diseases. Compartmentalization and trafficking of Treg and Th17 cells may be tissue- and/or organ-specific. This selective retention and trafficking is mediated by expression of distinct chemokine receptors and integrins. Upon trafficking to peripheral environments, the phenotype/development of T cell subsets (e.g., Treg, Th17 and Th1 cells) may be highly modulated, with environmental stimuli contributing to the plasticity of T cell development.

Dr. Zou reported on a poorly defined, minor IL-17^+^Foxp3^+^CD4^+ ^T cell population observed in human peripheral blood. This Treg subset selectively accumulates in the colitic microenvironment and associated colon carcinoma and has a phenotype and cytokine profile that overlaps with Th17 and Treg cells. Induction of these cells from memory CCR6^+ ^T or Treg cells is dependent on myeloid antigen presenting cells, IL-2 and TGFβ. This subset suppressed T cell activation and stimulated production of inflammatory cytokine in colitic tissues. Moreover, these cells strongly expressed IL-8 and promoted trafficking of neutrophils. Dr. Zou concluded that IL-8^+ ^and IL-17^+^Foxp3^+ ^cells may represent a unique "inflammatory" Treg population that contributes to the pathogenesis of ulcerative colitis, mechanistically linking chronic inflammation to development of colon cancer.

## Immune Cell Trafficking to Tumor Microenvironment

Incoming SITC president, Thomas F. Gajewski, MD, PhD (University of Chicago) and Elizabeth M. Jaffee, MD (Johns Hopkins University) co-chaired a session on trafficking of immune cells to the tumor microenvironment. Dr. Gajewski presented on regulation of anti-tumor immunity through migration of subsets of immune cells within the tumor microenvironment. Dr. Gajewski and colleagues have explored the relationship between the accumulation of CD8^+ ^T cells and the presence of immune inhibitory pathways within the tumor microenvironment. While it was generally assumed that the tumor establishes an immune suppressive microenvironment to inhibit infiltrating T cells, Dr. Gajewski has observed higher expression of immune inhibitory pathways in tumors that contain T cells. This has led to a new hypothesis in which the expression of IDO and PD-L1, and the accumulation of Tregs, is *dependent *upon the infiltration of CD8^+ ^T cells in the tumor site. These might be induced by specific factors produced by activated CD8^+ ^T cells.

A number of key factors in the tumor microenvironment associated with immune-mediated tumor control depend on regulated recruitment of subsets of inflammatory cells. This includes the priming phase (CD8α^+ ^DC recruitment), the effector phase (CD8^+ ^effector cell recruitment) and negative regulation (Treg recruitment). Understanding these factors should advance the development of new interventions to modify the microenvironment and better support T cell-mediated tumor rejection.

David W. Mullins, PhD (University of Virginia) discussed data demonstrating spatial and temporal regulation of CXCR3 chemokine production and CD8 T cell infiltration in the metastatic melanoma tumor microenvironment. Dr. Mullins reviewed results demonstrating that CXCR3 plays a key role in the infiltration of innate and adaptive effector cells into melanoma. Moreover, early-stage and late-stage tumors are differentially susceptible to infiltration and elimination by CD8^+ ^T effector cells due to temporal dysregulation of IFN-γ-induced chemokine production. He concluded that modulation of the tumor microenvironment to create and/or maintain a local chemotactic gradient may improve the therapeutic efficacy of cancer vaccines or adoptive transfer immunotherapies.

Elizabeth M. Jaffee, MD (Johns Hopkins University) presented on regulatory T cells and their role in the tumor microenvironment. Most T cell responses to antigen-specific cancer vaccines are weak and do not effectively control tumor growth. In many cases this may reflect T cell tolerance to the specific tumor antigen and highlights the need to better understand the context of tumor antigens to develop effective interventions that can reverse the tolerance and improve response of these T cells to the tumor. Dr. Jaffee and colleagues have described immune tolerance in the HER-2*/neu *transgenic (*neu*-N) mouse model of breast cancer and have used this model to explore mechanisms that suppress high avidity antigen-specific CD8^+ ^T cells. Dr. Jaffee and colleagues have previously reported that CD8^+ ^T cells specific for the immunodominant neu epitope, RNEU_420-429_, were observed only in *neu*-N mice that had been treated with cyclophosphamide + vaccine and rejected tumor challenge, but not in *neu*-N mice that had received vaccine alone. Moreover, high avidity RNEU_420-429_-specific CD8^+ ^T cells were also observed in vaccine-treated mice that had been previously depleted of CD25^+ ^Tregs. Dr. Jaffee's team has developed transgenic mice with high and low avidity TCRs specific for the same RNEU_420-429 _epitope. Using these mice, they evaluated differences in tumor-trafficking and function between high and low avidity cancer antigen-targeted T cells. Dr. Jaffee described a subset of Tregs that block trafficking and activation of high avidity T cells in *neu*-expressing tumors based on adoptive transfer of naïve T cells from these mice into tumor bearing *neu*-N mice.

Dr. Jaffee also described current cancer vaccine investigations of mesothelin as a T cell target in patients with pancreatic cancer. She reported that vaccination of pancreatic cancer patients with two genetically-modified pancreatic tumor cell lines induced T cell responses against new pancreatic tumor antigens. While elevated mesothelin expression by the tumor correlated with shorter DFS after surgery, mesothelin-specific T cell responses in 60 patients who received the vaccine after surgery corresponded to improved DFS.

Arianna Calcinotto (San Raffaele Scientific Institute) presented data demonstrating that NGR-TNF, a selective vessel-targeting agent, increases the therapeutic potential of chemo-immunotherapy. NGR-TNF is a novel hybrid molecule composed of TNF fused to NGR--a peptide with the capacity to target tumor blood vessels and to affect their physiology via CD13. Vascular targeting with this agent enhances the therapeutic index of active and adoptive immunotherapies. Results presented indicate that combination strategies incorporating active immunotherapy, vascular targeting with NGR-TNF and chemotherapy are synergistic in a mouse model of melanoma. NGR-TNF led to a temporal and selective activation of tumor endothelial cells that overexpressed adhesion molecules. Following treatment with NGR-TNF, activated CD8^+ ^T cells rapidly and selectively infiltrated the tumor, maintaining their effector functions within the tumor environment.

## iSBTc/SITC Biomarkers Task Force Update

Lisa H. Butterfield, PhD (University of Pittsburgh) provided an update on the 2009 iSBTc-FDA-NCI Workshop on Prognostic and Predictive Immunologic Biomarkers in Cancer. The program focused on the unique issues related to immunologic monitoring assays as well as novel methodologies for assessing the immune landscape in cancer. Standardization of assays, assay validation, and appropriate potency assays were the subject of one subcommittee, while the other assessed the clinical utility of promising novel technologies and discussed recommendations on how to incorporate them into the clinical arena. Since the 2009 Workshop the iSBTc Biomarkers Task Force has prepared a publication with recommendations [1] and the society hosted a Symposium on Immuno-Oncology Biomarkers on September 30, 2010 to explore issues related to biomarkers in cancer immunotherapy [2]. Presentation slides and other information about this Immuno-Oncology Biomarkers Symposium are available on the society's website [3].

## Cancer Immunotherapy Trials Network Update

At a special session, William Merritt, PhD (NCI) announced Martin "Mac" Cheever, MD (Fred Hutchinson Cancer Research Center, University of Washington) as the recipient of the U01 grant that will fund the Cancer Immunotherapy Trials Network (CITN). In addition to this first public announcement, Dr. Merritt discussed the application process for the clinical immunotherapy member sites. Dr. Cheever reviewed the vision for the CITN to provide a highly collaborative structure to efficiently develop innovative, intelligent and biologically dictated immunotherapy regimens. Dr. Cheever summarized the overall strategy of the network to design, develop and conduct important trials not otherwise possible. Under Dr. Cheever's leadership the CITN can be expected to 1) develop regimens that prospectively and predictably greatly increase the number of T cells specific for known and defined antigens; 2) develop "off the shelf" regimens that can be used by multiple investigators in multiple circumstances to serve as the backbone for further immunotherapy agent development; and 3) focus on agents, antigens and regimens that have received consensus prioritization in previous workshops.

## 25^th ^Anniversary and Awards

At its 25^th ^Anniversary and Awards Reception that was hosted at the Smithsonian National Museum of Natural History on October 3, 2010, iSBTc/SITC was proud to honor a number of leading research teams and individual investigators who have made significant contributions to the field of cancer immunotherapy. These accomplishments and awards have been previously summarized and are reviewed on the society's website [4].

## Adoptive T Cell Transfer: The Next Wave

Patrick Hwu, MD (University of Texas, MD Anderson Cancer Center) and Ton N. Schumacher, PhD (Netherlands Cancer Institute) co-chaired the final session on adoptive T cell transfer. Malcolm K. Brenner, MD, PhD (Baylor College of Medicine) presented on improving the efficacy and safety of genetically modified virus-specific T cells for solid tumors. Dr. Brenner demonstrated that EBV-specific CTLs can be modified to express chimeric antigen receptors (CAR) against solid tumors and that these CAR-CTLs can survive long term and produce clinical response in neuroblastoma, even in the absence of lymphoablation. Dr. Brenner discussed extending this approach beyond neuroblastoma. He summarized findings that indicate that safety of this approach may be enhanced by incorporation of the fast-acting suicide gene *icasp9*.

Sid Kerkar, MD (National Cancer Institute, Center for Cancer Research, NIH) described how functional reprogramming of the tumor stroma by IL-12-engineered T cells is required for anti-tumor immunity. Dr. Kerkar presented results from a mouse model that indicate that small numbers of tumor-specific T cells that overproduce IL-12 within the tumor microenvironment can eradicate large, established B16 melanoma tumors. Moreover, anti-tumor immunity was shown to be dependent on IL-12 and IFN-g-dependent sensitization of host bone marrow-derived myeloid cells. Further, Dr. Kerkar demonstrated that T cells engineered to express IL-12 induce functional changes in the myeloid cell populations that reside within tumors. Dr. Kerkar concluded that anti-tumor immunity appears largely dependent on the ability of endogenous cells to cross-present tumor antigens *in vivo*.

Pallavi Raja Manuri, PhD (University of Texas, MD Anderson Cancer Center) presented on noninvasive positron emission tomography (PET) imaging of *Sleeping Beauty *(SB) modified CD19-specific T cells expressing herpes simplex virus1-thymidine kinase (HSV1-tk). Dr. Manuri argued that to improve the design, application and evaluation of adoptive T cell therapy monitoring methods are required that can detect, locate and serially quantify the cell-mediated immune responses. While current monitoring methods (e.g., histology, flow cytometry, Q-PCR and/cytokine analysis) are generally invasive techniques, PET by contrast is noninvasive. PET can provide accurate, sensitive whole-body imaging and allows repetitive measurement *in vivo*. Dr. Manuri and colleagues achieved co-expression of a CD19-specific CAR and HSV1-tk by *SB *transposition. These *SB *modified CD19CAR^+^TK^+ ^T cells allowed spatio-temporal visualization by mPET using ^18^F-FEAU. Ablation of transferred T cells was achieved with ganciclovir. The cells were shown to have the capacity to kill CD19^+ ^tumor targets.

Ton N. Schumacher, PhD (Netherlands Cancer Institute) discussed dissection of therapy-induced melanoma-reactive cytotoxic T cell responses. Dr. Schumacher reported on the development and validation of technology for high-throughout detection of melanoma-specific T cell reactivity induced by immunotherapy. T cell responses in TIL cell products were found to be diverse, biased towards melanoma differentiation antigens (MDA) and C/G antigens, and highly variable between patients. Additionally, in 'young TIL', the individual T cell responses were generally of low magnitude. Dr. Schumacher noted that T cell responses in TIL cell products predict immune reactivity post-therapy. There was no evidence of epitope spreading.

Matthias Stephen, MD, PhD (Massachusetts Institute of Technology) discussed therapeutic cell engineering using surface-conjugated synthetic nanoparticles. Approaches to enhance persistence and functionality of transferred T cells are desired to improve adoptive cell therapies for cancer treatment. Dr. Stephen described an adoptive T cell therapy strategy that involved *ex vivo *conjugation of cytokine-loaded nanoparticles to T cells. These nanoparticles remained on the cell surface of the transferred T cells and did not interfere with trafficking to the tumor or effector cell functions. Upon transfer, T cells carrying the cytokine-loaded nanoparticles proliferated *in vivo *and provided a robust anti-tumor response. The cytokine-loaded nanoparticles provide a pseudo-autocrine stimulation of the transferred T cells, enabling the use of minimal cytokine doses that have no therapeutic effect when delivered systemically in a soluble form. Dr. Stephen reported that the strategy is generalizable for use with a wide range of cytoreagents to minimize systemic side effects.

Patrick Hwu, MD (University of Texas, MD Anderson Cancer Center) discussed results of an ongoing Phase II clinical trial of adoptive cell therapy with TIL in patients with metastatic melanoma regardless of HLA subtype. In this trial, autologous TIL were expanded and transferred back to the patient following lymphodepletion. Adoptive transfer was followed by two cycles of high dose IL-2 treatment. Thirty-one patients with stage IIIc-IV melanoma and a broad range of HLA subtypes were treated with this approach, infusing 8 x10^9 ^to 1.5 × 10^11 ^TILs. TILs were successfully expanded in 65% of cases and *in vitro *anti-tumor activity was observed in 70%. Clinical response (RECIST) was evaluated at weeks 6 and 12, and every 3 to 4 months thereafter. Dr. Hwu reported that of the 31 patients treated, 16 (52%) had a clinical response with one complete response and 15 partial responses. The clinical response correlated with the total number of TILs infused, with higher numbers associated with positive responses. Responders had higher numbers and longer persistence of dominant TCR clones. Dr. Hwu presented data that suggest emerging T cell clones may contribute to delayed clinical responses. The probability of a clinical response correlated with higher percent and number of CD8^+ ^TILs infused and with lower numbers CD4^+ ^TILs infused. Moreover, the differentiation status of the CD8^+ ^TILs infused correlated with clinical response, with positive responses correlating most strongly with CD8^+ ^T effectors cells (CD45RA^- ^CD62L^- ^CD27^-^). Interestingly, Dr. Hwu observed that expression of the negative costimulation molecule "B and T lymphocyte attenuator" (BTLA) on CD8^+ ^TIL was a better predictor of positive clinical response than PD-1. These observations led to the conclusion that the role of BTLA may depend on the context of expression and differ based on the differentiation state of the T cells. Dr. Hwu reported that there was no difference in relative telomere length of the infused TIL between responders and non-responders. He concluded that in this ongoing clinical trial, they have observed a high clinical response rate with adoptive cell therapy of metastatic melanoma. CD8^+ ^TILs appear essential in shrinking tumor, and TIL differentiation status and phenotype predict clinical response better than telomere length. Additionally, the observation that some patients experience delayed clinical responses following a period of stable disease suggests that additional therapies that compromise function or survival of T cells should not be performed until there is clear evidence of disease progression.

## Conclusion

In conclusion, the 25^th ^Annual Meeting of iSBTc/SITC included dynamic presentations, interactive panel discussions and scientific posters on timely topics in cancer immunotherapy as well as valuable information on clinical trial design and regulatory issues to advance collaboration and translation of cancer immunotherapies. In addition to the many exciting oral presentations, investigators from around the globe presented more than 180 posters detailing important scientific advances in various areas related to tumor immunology and cancer immunotherapy. These abstracts have been published and are available from the society's website, as are webinars of selected presentations of the scientific sessions [5]. With many recent advances in tumor immunology and cancer immunotherapy, as demonstrated by the innovative basic and translational research presented at this meeting, we look forward to the 26^th ^SITC Annual Meeting in North Bethesda, MD, November 3 - 6, 2011, which will explore the following topics: biology and application of dendritic cells; immunology of cancer stem cells and epithelial-to-mesenchymal transition (EMT); uncoupling negative regulation in the tumor microenvironment; genetically engineered receptors and adoptive cell therapies; characterization of inflammatory infiltrates in human cancers; state of the art animal models and veterinary applications for cancer and immunology; high throughput technologies for immune monitoring and prostate cancer as a learning model. Research will also be presented in poster format on the following topics: therapeutic monoclonal antibodies in cancer; immunotherapy combinations; innate immunity in cancer; tumor vasculature, chemokines and lymphocyte trafficking to the tumor; and targeted therapies and anti-tumor immunity. By promoting interaction and scientific exchange over these important topics, SITC strives to accelerate collaboration, research and clinical translation of immunotherapies that will improve the outcomes of patients with cancer.

## Competing interests

JB and FM declare that they have no competing interests. PK discussed αDC1s, which are the subject of a pending patent application. There are no active commercialization efforts and PK does not receive any royalties or other forms of remuneration related to this intellectual property. This situation is considered a minimal conflict of interest by the University of Pittsburgh. VS discloses the following relationships: Abbott, Consultant/Advisory Board receiving Consulting Fees/Honoraria; Bayer/Onyx, Consultant/Advisory Board receiving Consulting Fees/Honoraria; BMS/Medarex, Consultant/Advisory Board receiving Consulting Fees/Honoraria; Eli Lilly, Consultant/Advisory Board receiving Consulting Fees/Honoraria; Glaxo Smith-Kline/Synta, Consultant/Advisory Board receiving Consulting Fees/Honoraria; Merck(Schering), Speaker's Bureau receiving Honoraria; Merck (Schering), Consultant/Advisory Board receiving Consulting Fees/Honoraria; Pfizer, Consultant/Advisory Board receiving Consulting Fees/Honoraria. PC discloses the following relationship: Transgene, Advisory Committee receiving Honorarium. EJ discloses that The Johns Hopkins University, through a licensing agreement with BioSante, has the rights to future royalties on the GVAX approach discussed in this meeting. TG discloses the following relationships: GSK-Bio, Consultant and Protocol PI, receiving Honoraria and Clinical Trial Support; Eisai, Consultant and Protocol PI, receiving Honoraria and Clinical Trial Support; BMS, Consultant and Protocol PI, receiving Honoraria and Clinical Trial Support; Novartis, Protocol PI, receiving Clinical Trial Support; Roche, Protocol PI, receiving Clinical Trial Support.

## Authors' contributions

JB prepared the manuscript. PK, VS, PC and EJ co-organized the meeting and critically reviewed the manuscript. TG and FM reviewed the manuscript, are officers of the Society for Immunotherapy, and serve on the society's Scientific Program Committee, which reviewed and approved the Annual Meeting program. All authors read and approved the final manuscript.
